# Supplementary material for: Altered immunity to microbiota, B cell activation and depleted γδ/resident memory T cells in colorectal cancer
Source: Cancer Immunol Immunother. 2022 Mar 22;71(11):2619–29. doi: 10.1007/s00262-021-03135-8 (PMC9519644; doi:10.1007/s00262-021-03135-8)
Supplement: Supplementary file 1 — Supplementary file1 (PDF 99 KB) [file 262_2021_3135_MOESM1_ESM.pdf]

Supplementary Table 1. Antibodies used:

ANTIGEN	FLUOROCHROME	mAb CLONE	SUPPLIER
CD4	BB515	SK3	BD Biosciences
CD8α	BV605	SK1	BioLegend
CD19	PE-Cy5	HIB19	BD Biosciences
CD24	APC-Cy7	ML5	BioLegend
CD27	PE	M-T271	BD Biosciences
CD38	BV605	HIT2	BioLegend
CD39	PE-Cy7	A1	BioLegend
CD69	BV605	FN50	BioLegend
CD73	APC	AD2	BioLegend
CD103 (integrin αE)	PE	Ber-ACT8	BioLegend
IgA	APC	IS11-8E10	Miltenyi Biotec
IgD	PE-Cy7	IA6-2	BD Biosciences
IgG	FITC	IS11-3B2.2.3	Miltenyi Biotec
IgM	BV421	G20-127	BD Biosciences
Integrin β7	PE	FIB504	BD Biosciences
IFN-γ	FITC	B27	BD Biosciences
Runx3	PE	R3-5G4	BD Biosciences
TCR γδ	FITC	11F2	BD Biosciences
TNF-α	PE-Cy7	MAb11	eBioscience
